# Effect of the Interaction of Veratrum Nigrum with Panax Ginseng on Estrogenic Activity *In Vivo* and *In Vitro*

**DOI:** 10.1038/srep26924

**Published:** 2016-05-27

**Authors:** Ying Xu, Jie Ding, Jin-na An, Ya-kun Qu, Xin Li, Xiao-ping Ma, Yi-min Zhang, Guo-jing Dai, Na Lin

**Affiliations:** 1 Institute of Chinese Materia Medica, China Academy of Chinese Medical Sciences, Dongcheng District Dongzhimen Nanxiao Road 16, Beijing 100700, China; 2 Institute of Chinese Materia Medica, Shanghai University of Traditional Chinese Medicine, Pudong District cailu Road 1200, Shanghai 100101, China

## Abstract

Panax ginseng (GS) and Veratrum nigrum (VN) are representative of incompatible pairs in “eighteen antagonistic medicaments” that have been recorded in the Chinese medicinal literature for over 2,000 years. However, evidence linking interference effects with combination use is scare. Based on the estrogen-like effect of GS described in our previous studies, we undertake a characterization of the interaction on estrogenic activity of GS and VN using *in vivo* models of immature and ovariectomized (OVX) mice and *in vitro* studies with MCF-7 cells for further mechanism. VN decreased the estrogenic efficacy of GS on promoting the development of the uterus and vagina in immature mice, and reversing the atrophy of reproductive tissues in OVX mice. VN interfered with the estrogenic efficacy of GS by decreasing the increase of the serum estradiol and the up-regulation of ERα and ERβ expressions by treatment with GS. And VN antagonized the estrogenic efficacy of GS on promoting the viability of MCF-7 cells and up-regulation of protein and gene expressions of ERs. In conclusion, this study provided evidence that GS and VN decreased effects on estrogenic activity, which might be related to regulation of estrogen secretion and ERs.

In different regions and cultures, herbal products are used as single herb, combination of herbs, or combination of herb(s) and drug(s). When herbs are used in combination, the effects can be complicated because various interactions can occur among the individual components^1^. Herb-herb combinations have been used in Chinese medicine for thousands of years. Incompatibility is one of the basic modes of herb-herb interaction. “The eighteen incompatible medicaments” -the controversial prohibited combinations in TCM, which suggests drugs in the eighteen incompatible medicaments should not be used together because interaction leads to an unexpected consequence^2^. However, even today, less evidence links interference effects with combination use. Moreover, some studies have used combinations of drugs in “the eighteen incompatible medicaments” to treat incurable diseases^3^. Therefore, it is important to determine whether these agents are incompatible when used in combination, and the reasons for any incompatibility.

Panax ginseng (GS) and Veratrum nigrum (VN) are representative of incompatible pairs recorded in “the eighteen incompatible medicaments”. Currently, researchers focus primarily on the toxicological response of the GS-VN combination. However, it is worth noting that according to the description of GS-VN incompatibility in the Chinese medicinal literature, VN could reduce the effect of GS in a certain ratio when used in combination.

GS is one of the traditional Chinese medicines being widely studied in the West^4^. GS has been used for over 2000 years for treating various illnesses, such as antitumor, antistress, and antioxi-dant activities^5^. In recent years, ginseng has become one of the mostly used alternative medicines for hormone replacement therapy as it was shown to possess estrogen-like activity^6^.

VN is commonly known as Black False Hellebore and highly poisonous perennial herb native to Asia and Europe^7^. VN has ability to cause nausea and vomiting, it is applied to dyspnea in epilepsy or stroke patients^8^. Studies have revealed that VN can decrease blood pressure and heart rate in hypertensive rats^9^, and it affords significant protection against hepatic ischemia/reperfusion injury in rats^10^.

The exact pharmacological mechanisms of the interactions of GS-VN are not clearly understood. There are few researches that focus on the effects of incompatible pairs. GS has been observed to suppress the enzymatic activity and mRNA expression of CYP450 isozymes in the presence of VN^11^. Another study showed that GS-VN in combination exerted anti-obesity effects both *in vivo* and *in vitro*. GS-VN in combination also significantly reduced weight gain and the fat pad weight in high fat-induced obese-mice. The weight gain in the GS-VN at 1:1 ratio combination group was also significantly decreased compared with the GS only group^5^. In our previous study, VN attenuated the anti-fatigue effect of GS in normal mice, which indicates the efficacy of interference for GS-VN used in combination^12^. The results of different research are inconsistent. In our studies, we have provided systematic evidences that GS exerts estrogenic effects in immature mice or ovariectomized (OVX) mice^13^,^14^. This led us to consider the following question: as incompatible pairs, does VN have anti-estrogenic effects? In order to answer this question, this study concentrated on the interaction on estrogenic activity of GS after treated with VN using *in vivo* model of immature and ovariectomized (OVX) mice. *In vitro* studies with the MCF-7 cells were also performed to obtain further information on the molecular mechanism. This study is part of an on-going effort to provide insight into the nature of GS and VN incompatibility.

## Results

### Effect of incompatibility of VN and GS on body, uterine and adrenal gland weights

**Figure 1:**
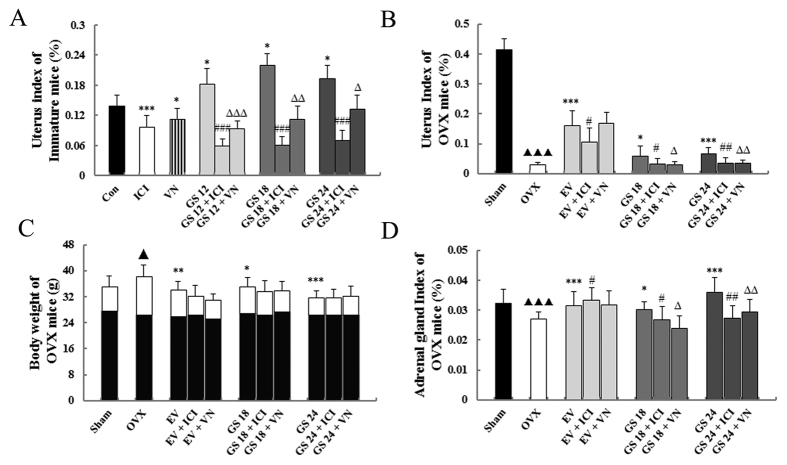
The effects of incompatibility of Veratrum nigrum (VN) and Panax ginseng (GS) on uterine, adrenal gland and body weights. (**A**) The uterine weights of immature mice were measured at the end of the 7-day treatment period. (**B**) The uterus index for ovariectomized (OVX) mice was measured at the end of the 4-week treatment period. (**C**) Body weights of OVX mice were measured once per week for 4 weeks. (**D**) The adrenal gland index of ovariectomized (OVX) mice was measured at the end of the 4-week treatment period. Data are the mean and standard deviation (SD) of samples from 10 mice. *P* values are for the one-way analysis of variance (ANOVA) comparing the treatment group with untreated mice. ***P < 0.001, **P < 0.01 and *P < 0.05 compared with the Con or OVX group; ^###^*P* < 0.001 and ^#^*P* < 0.05 compared with the GS group or EV group; ^∆∆∆^*P* < 0.001, ^∆∆^*P* < 0.01, and ^∆^*P* < 0.05 compared with the GS group or EV group; ^▴▴^*P* < 0.01 and ^▴^*P* < 0.05, compared with the Sham group.

Figure 1A,B showed that treatment with GS resulted in significant estrogenic activity. GS had modest stimulatory effects on the uterine weights of immature and ovariectomized (OVX) mice (all *P* < 0.05 or 0.001). GS+ ICI182, 780 (an estrogen antagonist) or GS+ VN induced a lower uterus index in immature and OVX mice compared with GS treatment alone (all P < 0.05, 0.01 or 0.001). The results suggest that VN could antagonize the estrogenic efficacy of GS on increasing the uterine weight of immature and OVX mice. VN showed activity comparable to the estrogen antagonist ICI on decreasing the uterine weights of mice.

Estradiol valerate (EV) and GS treatment significantly increased adrenal gland weight. GS had a trend of increasing weight with an increasing dose (*P* < 0.05 or 0.001) compared with untreated OVX mice. ICI and VN could attenuate the increases in adrenal gland weight resulted by GS treatment (all *P* < 0.05 or 0.01). However, VN could not inhibit the effects of EV treatment (Fig. 1D).

The OVX mice from all groups had similar initial mean body weights. At the end of the study, the mean body weight of mice in the OVX group was significantly higher than that of the sham group. EV and GS treatment significantly prevented the increase in body weight associated with estrogen (E_2_) deficiency due to ovariectomized operation. The estrogen antagonist ICI and VN could lower the estrogenic efficacy of EV or GS on preventing the increase in body weight of OVX mice, but these differences were not significant (Fig. 1D).

### Effect of incompatibility of VN and GS on levels of serum E_2_, luteinizing hormone and follicle-stimulating hormone

**Figure 2:**
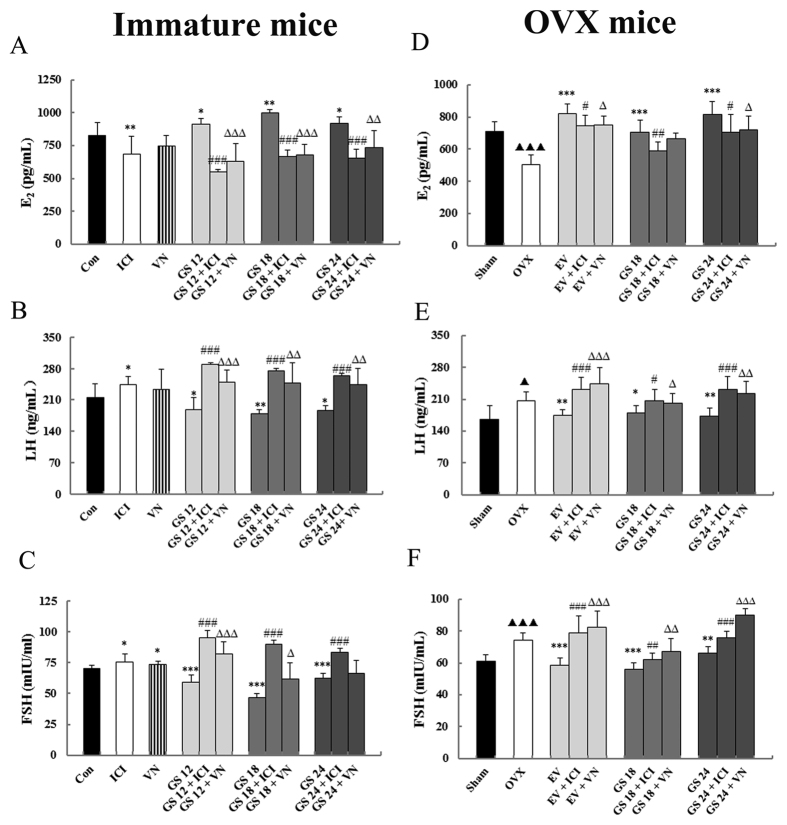
The effects of incompatibility of Veratrum nigrum (VN) and Panax ginseng (GS) on serum estradiol (E_2_), luteinizing hormone (LH) and follicle-stimulating hormone (FSH) in immature and ovariectomized (OVX) mice. Serum levels of E_2_ (**A**), LH (**B**) and FSH (**C**) from immature mice and serum levels of E_2_ (**D**), LH (**E**) and FSH (**F**) from ovariectomized (OVX) mice were measured at the end of the treatment period. Data are the mean and standard deviation (SD) of samples from 10 mice. *P* values are for the one-way analysis of variance (ANOVA) comparing treatment groups with untreated mice. ***P < 0.001, **P < 0.01 and *P < 0.05 compared with the Con or OVX group; ^###^*P* < 0.001 and ^#^*P* < 0.05 compared with the GS group or EV group; ^∆∆∆^*P* < 0.001, ^∆∆^*P* < 0.01 and ^∆^*P* < 0.05 compared with the GS group or EV group; ^▴▴▴^*P* < 0.01, ^▴▴^*P* < 0.01 and ^▴^*P* < 0.05 compared with the Sham group.

Immature and OVX mice are expected to have lower levels of serum E_2_ and increased levels of luteinizing hormone (LH) and follicle-stimulating hormone (FSH) compared with mature and sham-operated mice. Treatment with GS or EV significantly raised levels of circulating E_2_ compared to those of untreated immature and OVX mice (*P* < 0.05, 0.01 or 0.001). GS treatment significantly down-regulated LH and FSH content (*P* < 0.05, 0.01 or 0.001) in immature and OVX mice. VN significantly attenuated the increase of the serum E_2_ and down-regulation of LH and FSH in immature and OVX mice treated with GS. This effect was comparable to that of the estrogen receptor antagonist ICI (*P* < 0.05, 0.01 or 0.001). These results are depicted in Fig. 2.

### Effect of incompatibility of VN and GS on histology of the uterus and vagina

**Figure 3:**
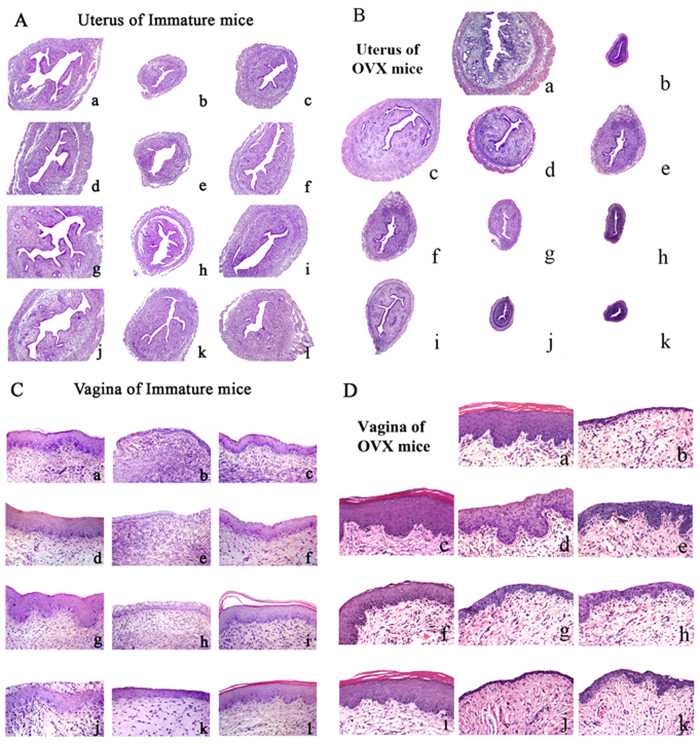
The effects of incompatibility of Veratrum nigrum (VN) and Panax ginseng (GS) on the histology of the uterus and vagina in immature and ovariectomized (OVX) mice. Representative photomicrographs taken at 200-X magnification of uterine and 400-X magnification of vaginal sections. (**A**,**B**) are the histology of the uterus in immature mice and ovariectomized (OVX) mice. (**C**,**D**) are the histology of the vagina in immature mice and ovariectomized (OVX) mice. The treatment groups in immature mice are shown: (a) control group; (b) treated with ICI; (c) treated with VN at 0.045 g/kg; (d) treated with GS at 12 g/kg; (e) treated with GS at 12 g/kg and ICI, (f) treated with GS at 12 g/kg and VN. (g) treated with GS at 18 g/kg; (h) treated with GS at 18 g/kg and ICI; (i) treated with GS at 18 g/kg and VN; (j) treated with GS at 24 g/kg; (k) treated with GS at 24 g/kg and ICI; (l) treated with GS at GS 24 g/kg and VN in immature mice. The treatment groups in OVX mice are shown: (a) sham-operated mice; (b) untreated OVX mice; (c) treated with EV; (d) treated with EV and ICI, (e) treated with EV and VN; (f) treated with GS at 18 g/kg; (g) treated with GS at 18 g/kg and ICI, (h) treated with GS 18 at g/kg and VN; (i) treated with GS at 24 g/kg; (j) treated with GS at 24 g/kg and ICI; and (k) treated with GS at 24 g/kg and VN in OVX mice.

Histological analysis of uterine sections revealed that treatment with EV or GS at any dose substantially induced the growth and development of the uterus in immature mice and restored the atrophy of the uterus in OVX mice compared with untreated controls. These results in treated samples were indicated by the thickening of the uterine endometrium, an increased number of glands and more extended glandular cavities compared with untreated samples. VN reduced the estrogenic efficacy of GS on promoting the growth and development of the uterus in immature mice and restoring the atrophy of the uterus in OVX mice. In GS + VN or ICI groups, the uterine morphology was atrophic, as indicated by the thinner uterine endometrium, the decreased number of glands, and fewer extended glandular cavities compared with samples treated with GS alone treatment. These results are depicted in Fig. 3A,B and Tables 1 and 2.

Figure 3C,D and Tables 1 and 2 also shows microscopic preparations of representative vaginas from one animal per treatment group. The EV treatment group displayed typical squamous multilayered epithelium with cornification in OVX mice. Treatment with GS at any dose increased epithelial thickness and the number of cell layers in both mouse models. Co-treatment of GS with VN or ICI resulted in vaginal epithelium of immature and OVX mice that were composed of fewer cell layers and flattened cells with less cornification when compared with GS treatment alone. VN showed activity comparable to ICI on delaying the growth and development of the uterus and vagina in immature and OVX mice.

Taken together, these studies provide evidence that VN decreased the estrogenic efficacy of GS on promoting development of the uterus and vagina in immature mice. VN also reversed the atrophy of the uterus and vagina in OVX mice and was comparable to the estrogen antagonist, ICI. These data prompted further studies to elucidate the molecular basis of incompatibility of VN and GS.

### Effect of incompatibility of VN and GS on expression of ER subtype in the uterus and vagina

**Figure 4:**
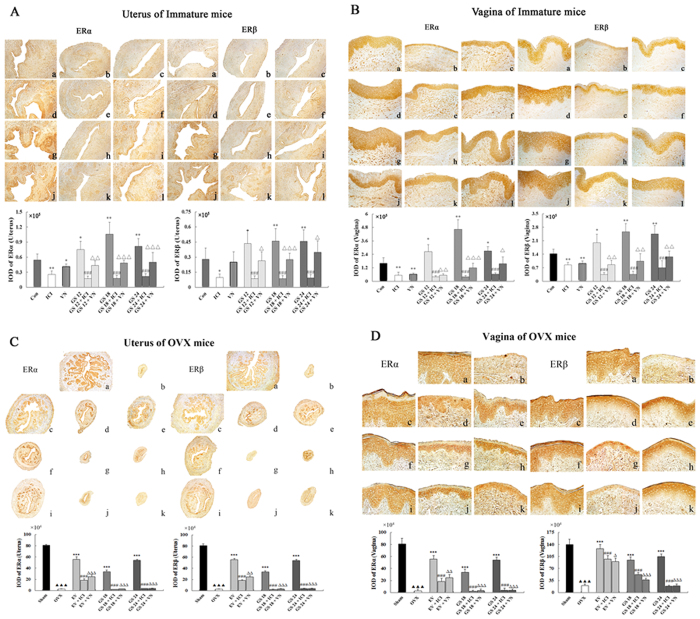
The effects of incompatibility of Veratrum nigrum (VN) and Panax ginseng (GS) on the expressions of estrogen receptor (ER) α and β in the uterus and vagina. ERs expressions were assessed by immunohistochemistry. Representative photomicrographs taken at 200-X magnification of uterine and 400-X magnification of vaginal sections. (**A**,**B**) show expression of ERs in immature mice. Treatment groups are shown: (a) control group; (b) treated with ICI; (c) treated with VN at 0.045 g/kg; (d) treated with GS at 12 g/kg; (e) treated with GS at 12 g/kg and ICI; (f) treated with GS at 12 g/kg and VN; (g) treated with GS at 18 g/kg; (h) treated with GS at 18 g/kg and ICI; (i) treated with GS at 18 g/kg and VN; (j) treated with GS at 24 g/kg; (k) treated with GS at 24 g/kg and ICI; and (l) treated with GS at 24 g/kg and VN. (**C**,**D**) show the expression of ERs in the ovariectomized (OVX) mice. Treatment groups are shown: (a) sham-operated mice; (b) untreated OVX mice; (c) treated with EV; (d) treated with EV and ICI, (e) treated with EV and VN;(f) treated with GS at 18 g/kg; (g) treated with GS at 18 g/kg and ICI, (h) treated with GS at 18 g/kg and VN; (i) treated with GS at 24 g/kg; (j) treated with GS at 24 g/kg and ICI; and (k) treated with GS at 24 g/kg and VN. Data are the mean and standard deviation from 10 mice. *P* values are for the one-way analysis of variance comparing the treatment group with untreated mice. ***P < 0.001, **P < 0.01 and *P < 0.05 compared with the Con or OVX group; ^###^*P* < 0.001, ^##^*P* < 0.01 and ^#^*P* < 0.05 compared with the GS group or EV group; ^∆∆∆^*P* < 0.001, ^∆∆^*P* < 0.01 and ^∆^*P* < 0.05 compared with the GS group or EV group; ^▴▴▴^*P* < 0.001, ^▴▴^*P* < 0.01, and ^▴^*P* < 0.05 compared with the Sham group.

Representative sections of the expression of estrogen receptor subtypes α and β (ERα and ERβ) in the uterus and vagina from each group and quantitative analysis are shown in Fig. 4. The expression of ERα and ERβ in the uterus and vagina after treatment with GS at any dose were significantly increased compared with the untreated group in immature (*P* < 0.05 or *P* < 0.001) or OVX mice (*P* < 0.001), respectively. ERs in the uterus were expressed in similar cell types in the GS-treated, EV-treated or sham-operated groups. These cell types were the epithelial cells of the endometrium, interstitial cells and smooth muscle cells. ERs in vagina were expressed in the vaginal epithelium cells of squamous and smooth muscle cells.

GS + VN or GS+ICI treatment induced clear and comparable down-regulation of ERα and ERβ in the reproductive tissues of immature and OVX mice compared with GS treatment alone. VN had a similar ability as the estrogen antagonist ICI to down-regulate the estrogenic efficacy of GS on decreasing the expression of ERs in the uterus and vagina. These data indicated that VN decreased the estrogenic efficacy of GS *in vivo* through ERs.

### Effect of incompatibility of VN and GS on protein and gene levels of ER subtype in the uterus and vagina

**Figure 5:**
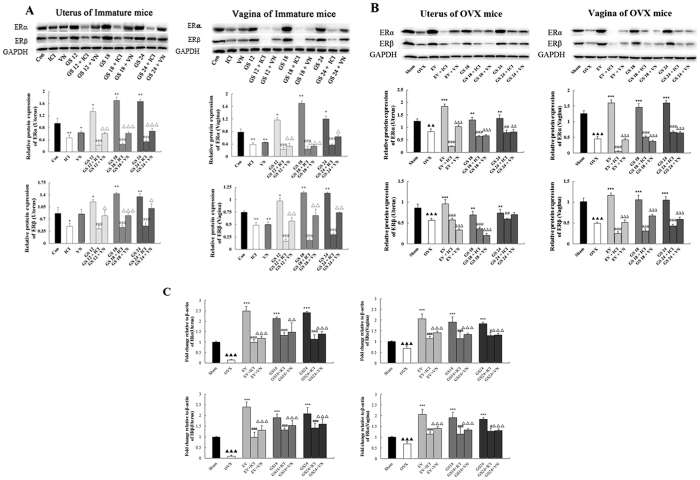
The effects of incompatibility of Veratrum nigrum (VN) and Panax ginseng (GS) on the protein or gene expression of estrogen receptor (ER) α and ERβ in the uterus and vagina of mice. Western-blot and Realtime PCR analysis was carried out as described in the Methods. Representative blots are shown above, and quantitative analyses are shown below. *P* values are for one-way analysis of variance (ANOVA) comparing treatment groups with untreated mice. ***P < 0.001, **P < 0.01 and *P < 0.05 compared with the Con or OVX group; ^###^*P* < 0.001, ^##^*P* < 0.01 and ^#^*P* < 0.05 compared with the GS group or EV group; ^∆∆∆^*P* < 0.001, ^∆∆^*P* < 0.01 and ^∆^*P* < 0.05 compared with the GS group or EV group; ^▴▴▴^*P* < 0.001, ^▴▴^*P* < 0.01, and ^▴^*P* < 0.05 compared with the Sham group.

Further evidence for the compatibility of the VN and GS was sought by determining the effects on protein and mRNA levels in target tissues by western blot and real-time quantitative PCR. The results are shown in Fig. 5A–C, compared with the untreated group, treatment with either EV or GS at any dose induced significant up-regulation of protein levels of ERα and ERβ in the reproductive tissues of immature or OVX mice, and also up-regulation of gene expression of ERα and ERβ in OVX mice (P < 0.05, 0.01 or P < 0.01). GS +VT or ICI in combination induced clear and comparable down-regulation of ERα and ERβ on protein levels in the reproductive tissues of immature and OVX mice, and also down-regulation of gene expression of ERα and ERβ in OVX mice when compared with GS treatment alone (P < 0.05, 0.01 or P < 0.01). VN showed activity comparable to ICI for decreasing the estrogenic efficacy of GS on decreasing the protein and genelevels of ERs in the uterus and vagina.

Taken together, these data provide further evidence that VN antagonism of the estrogenic efficacy of GS *in vivo* through ERs was comparable to that of the estrogen antagonist, ICI.

### Effect of incompatibility of VN and GS on viability of MCF-7, T47D and MDA-MA-231 cells

**Figure 6:**
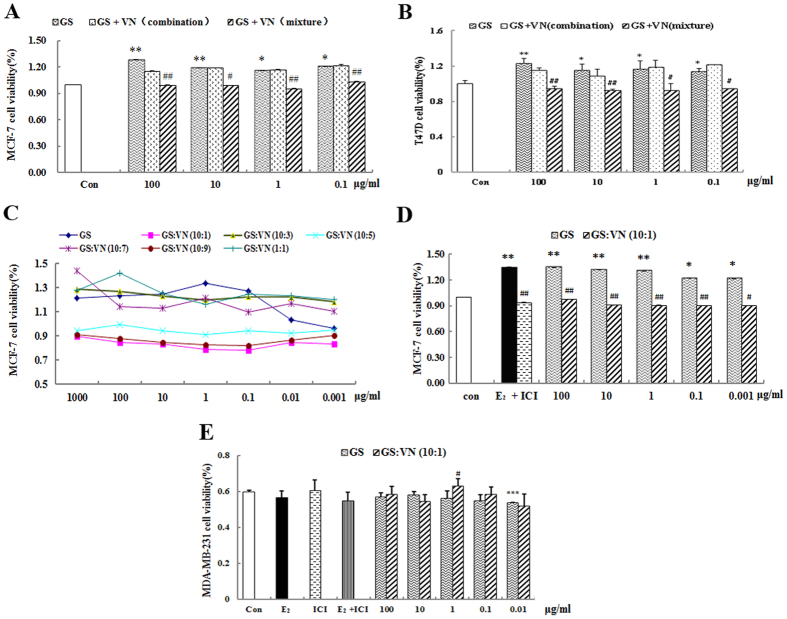
Effect of incompatibility of Veratrum nigrum (VN) and Panax ginseng (GS) on viability of MCF-7 cells, T-47D cells and MDA-MB231 cells. (**A**) Proliferation of MCF-7 cells for different methods of administration. (**B**) Proliferation of T-47D cells for different methods of administration. (**C**) Proliferation of MCF-7 cells in different compatibility proportions. (**D**) Effect of VN and GS on proliferation of MCF-7 cells. (**E**) Effect of VN and GS on the viability of MDA-MB-231 cells. Cell proliferation was carried out as described in the Methods. Results are expressed relative to growth of cells treated with 0.1% DMSO. Data are the mean and standard deviation of quadruplicate analyses, expressed relative to that of treatment with 0.1% DMSO. *P < 0.05 and **P < 0.01 compared with DMSO; ^#^*P* < 0.05 and ^##^*P* < 0.01 compared with GS or 0.01 mM E_2_.

In order to investigate the effect of incompatibility of VN and GS in more detail, we used the ER-positive and ER-negative human breast cancer cells, MCF-7, T47D cells and MDA-MB-231 cells as models. GS and VN were separately decocted and mixed (GS + VN mixture) or decocted together (GS + VN combination) and used to assess the effect on MCF-7 and T47D cells proliferation. Treatment with GS at dose levels of 0.1~100 μg/mL both stimulated proliferation of MCF-7 and T47D cells, demonstrating estrogenic activity in the GS extracts. The GS + VN mixture inhibited the proliferation of MCF-7 and T47D cells compared with GS treatment alone and resulted in significant differences (both *P* < 0.01). In contrast, GS + VN in combination did not display inhibited activity compared with GS treatment alone (Fig. 6A,B). Moreover, the maximum inhibition activity on MCF-7 cell was found in the compatibility proportion of 10:1 among the GS +VN mixture groups (Fig. 6C). VN at dose levels of 0.0001~10 μg/mL and GS +VN in the compatibility proportion of 10:1 significantly inhibited MCF-7 cells proliferation compared with GS alone (all *P* < 0.01). This result is comparable to that observed with ICI (Fig. 6D). However, E_2_, GS treatment alone or GS + VN mixture use at the compatibility proportion of 10:1 do not affect the ER-negative MDA-MB-231cell viability compared with DMSO treatment (Fig. 6E). These data prompted further studies to elucidate the molecular basis of incompatibility of VN-GS in MCF-7 cells.

### Effect of incompatibility of VN and GS on protein and gene levels of ER subtype in MCF-7 cells

**Figure 7:**
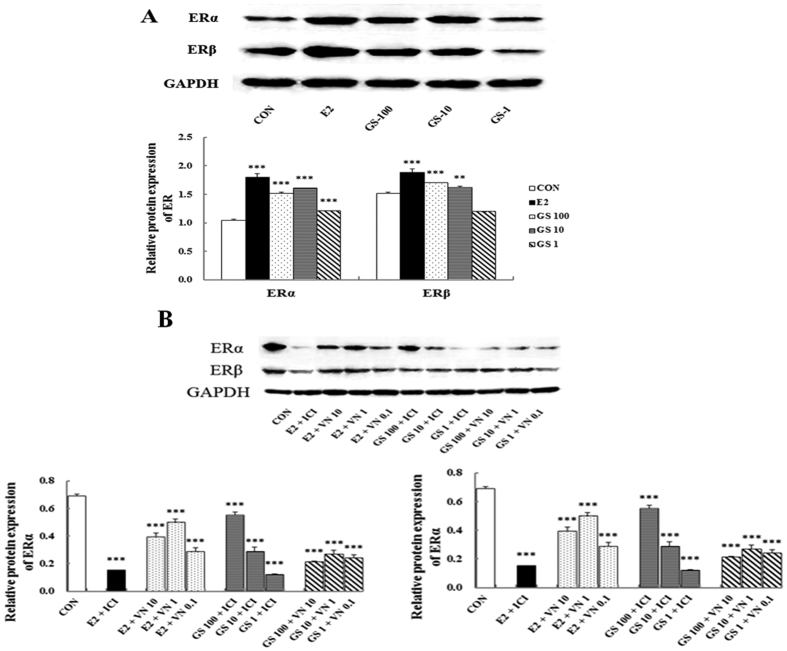
The protein expression of estrogen receptor (ER) α and ER β in MCF-7 cells. Western blot analysis of ERs expressions in MCF-7 cells was carried out as described in the Methods. *P < 0.05 and **P < 0.01, compared with DMSO.

**Figure 8:**
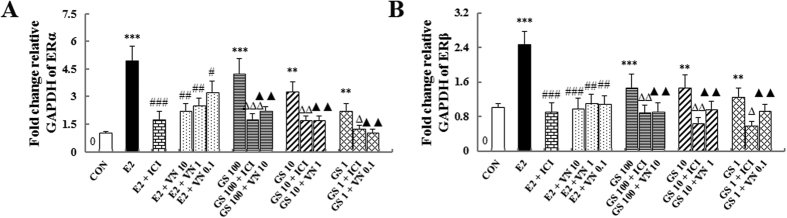
The gene expressions of estrogen receptor (ER) α and ER β in MCF-7 cells. Real-time PCR analysis was carried out as described in the Methods. ***P < 0.001, **P < 0.01, and *P < 0.05 compared with the control group; ^###^*P* < 0.001 and ^#^*P* < 0.05 compared with the E_2_ group; ^∆∆∆^*P* < 0.001, ^∆∆^*P* < 0.01, and ^∆^*P* < 0.05 compared with the GS group; ^▴▴^*P* < 0.01 and ^▴^*P* < 0.05 compared with the GS group.

Further evidence for the incompatibility of VN and GS was sought by determining the effects on the protein and mRNA levels of ER subtype in MCF-7 cells by western blots and real-time quantitative PCR. The results are shown in Fig. 7A, compared with the untreated group, treatment with either 17β-estradiol or GS induced significant up-regulation of ERα and ERβ protein and gene expression in MCF-7 cells (all *P* < 0.001). GS + VN or GS+ICI mixtures induced clear and comparable down-regulation of ERα and ERβ protein and gene expression levels when compared with GS treatment alone (Figs 7B and 8). VN demonstrated an ability similar to ICI for down-regulating the estrogenic efficacy of GS on increasing the protein and gene expression of ERs in MCF-7 cells.

Taken together, these studies provide evidence that VN interfered with the estrogenic efficacy of GS *in vitro* through ERs. The effects of VN were comparable to that of the estrogen antagonist, ICI.

## Discussion

The rational use and safety is a key both in traditional Chinese medicine and west medicine research field. Currently, little evidence links interference effects with GS -VN in combination use. This study aimed to evaluate the antagonistic effects of VN against the estrogen-like effects of GS using *in vivo* and *in vitro* assays. The results showed that VN exhibits antagonistic features against the estrogenic efficacy of GS by attenuating the increase of serum estradiol and the up-regulation of ERα and ERβ expression caused by treatment with GS. It suggested that GS and VN are mutually incompatible and therefore using these together in prescriptions should be avoided.

The doses of GS and VN selected were based on previous studies^13^,^14^. Our preliminary experimental results demonstrate that GS exerts estrogenic activity at a daily dose of 12.0, 18.0 and 24.0 g/kg in a physiological model of immature mice after short-term administration. The dose dependency of some responses suggests that the range of doses could have been extended to higher doses in a pathological model of OVX mice after long-term administration. The lowest dose of GS 12 g/kg did not increase the uterine weights and serum estradiol levels, but did up-regulate the expression of estrogen receptors in OVX mice. Hence, the daily dose of GS selected in the present study was 12.0, 18.0 and 24.0 g/kg in immature mice and 18.0 and 24.0 g/kg in OVX mice. In our previous immature mice research, the lower dose of VN of 0.045 g/kg, which was based on a low recommended dose of 0.005 g/kg in humans according to “Zhong Hua Ben Cao”, induced the more profound response on decreasing the uterine weights and serum E_2_ of immature mice. The higher dose of VN of 0.09 g/kg, based on a recommended high dose of 0.01 g/kg in humans did not exert any response (Fig. S1). VN is highly poisonous perennial herb and safe dose range is very narrow. 0.06 g/kg could induce minor toxic on mice, and main toxic reactions are shaking, twitching, and convulsions^15^. Therefore, we choose the effective doses of the two herbs on playing estrogenic/ anti-estrogenic activity to address why combinatory treatment of VN and GS has been avoided historically in the Chinese medicine practice.

Herb-herb interaction leading to an unfavourable outcome is the situation in which a combination results in toxic or severe adverse effects. It has been reported that a GS-VN combination has a better inhibitive effect on the growth of HepG2 cells than VN dose^16^. GS has been observed to suppress the enzymatic activity and mRNA expression of CYP450 isozymes in the presence of VN^11^. Another situation of incompatibility occurs when the therapeutic effect of one herb is diminished by another herb. This can be interpreted as an antagonistic interaction. In our previous study, VN weakened the anti-fatigue effect of GS in normal mice, which indicated efficacy of interference for the GS-VN combination^12^. In the dosage range of the Chinese Pharmacopoeia, VN could decreases, and even counteract the effects of GS on strengthening body resistance, the degree of this effect changes with ratio and dose^17^.

To further confirm whether GS and VN are incompatible, *in vivo* and *in vitro* assays were used and were based on the estrogen-like effect of GS. As a gold standard of estrogenic activity, *in vivo* study was used to determine that the uterine weights of mice treated with VN were much lower than GS treatment groups. Moreover, VN antagonized the estrogenic efficacy of GS on promoting development of the uterus and vagina in immature mice, and reversing the atrophy of the uterus and vagina in OVX mice. This was indicated by obvious degeneration of the cavities, endometrium and secretory glands in the uterus, and the presence of fewer cell layers in the vagina.

Our *in vitro* results are also consistent with the *in vivo* results. GS induced the proliferation of MCF-7 and T47D Cells, which are positive for the ER. The GS +VN mixture inhibited the proliferation of MCF-7 and T47D cells compared with GS treatment alone. However, GS treatment alone or GS +VN mixture use at the compatibility proportion of 10:1 do not affect the ER-negative MDA-MB-231cell viability compared with DMSO treatment (Fig. 6E). These results showed GS and VN decreased the effects on estrogenic activity maybe relate to ERs. Moreover, the antagonistic effect of GS + VN mixture (combination with single-fried water extract) was better than GS + VN combination (co-fried water extract). It is worthy of mention that the antagonistic effect was most significant when the compatibility proportion between GS and VN was 10:1, and the ratio is the same ratio in which GS and VN interaction induced the maximum opposite effects recorded in the traditional and modern literature. These results reveal that VN does antagonize the estrogenic efficacy of GS under certain conditions or dosage ranges. Interestingly, our results are consistent with the records of the ancient famous medical books. Based on Chinese herbal medicine theory and practice, VN as an emetic can strongly irritate the gastric mucosa. GS as a tonic medicine is traditionally used alone and is unsuitable for use with the emetic.

The biological effects of GS depend on not only the level of estrogen but also on the distribution and expression levels of the corresponding ERs in the target organs and cells^18^,^19^,^20^. To determine the exact pharmacological mechanisms of such interactions between GS and VN, the serum hormone levels, the protein and mRNA expression of ERα and ERβ were detected. VN attenuated the increase of serum estradiol and the up-regulation of ERα and ERβ protein and mRNA expressions in the reproductive tissues of immature or OVX mice treated with GS. Estrogens tightly regulate cell proliferation and differentiation particularly in the uterus, vagina and mammary gland of the female reproductive tracts. ERα action is essential for induction of uterine muscular disorganization and vaginal epithelial cell proliferation caused by estrogens during the critical developmental stage^21^,^22^. These foundational effects on ERα protein and mRNA expression may explain the effect of VN, GS, and GS-VN in combination on the atrophy of the uterus and vagina.

VN significantly decreased adrenal gland weight and increased circulating FSH and LH. When used in the presence of VN, the up-regulating effect of GS on the expression levels of protein and gene in MCF-7 cells greatly reduced or even eliminated. This activity is similar to the estrogen receptor antagonist ICI. These results demonstrate that the antagonistic activity of VN is mediated by stimulating the biosynthesis of estrogen in circulation and decreasing the quantity of ERs in the target organs and cells.

Recent reports indicate that ginsenosides, especially their aglycone constituent, are structurally similar to several steroids, including female hormones. As such, they are effective components of the estrogen-like activity of GS^23^,^24^,^25^,^26^. Changes in the chemical component between GS + VN and single herb were detected with the technology of chemical fingerprinting using the UPLC/TOF-MS platform. This revealed that the contents of ginsenosides significantly decreased and the contents of veratrum alkaloids increased when GS used in the present of VN^27^,^28^. Veratrum alkaloids, such as veratramine, jervine, veratrosine, veratrum alkamine and germidine, were assumed to contribute to the toxic effect of GS and VN, because of the change tendency is same with the death rate of mice^27^. Interestingly, the chemical structure of most of veratrum alkaloids is similar to steroids. Our study suggest that VN is a potential antagonist of estrogen receptors. Although the exact effective components remain unknown, this finding encourages us to do further research on VN.

## Conclusion

This is the first report of estrogenic activity on incompatible pairs, Panax ginseng and Veratrum nigrum. These findings provide systematic evidence that VN interferes with the estrogenic efficacy of GS by attenuating the increase of serum estradiol and the up-regulation of ERα and ERβ expression from treatment with GS. Based on the theory of traditional Chinese medicine, our result proved that GS is unsuitable for use with VN. This also provides pharmacological evidence for the eighteen incompatible medicaments.

## Methods

The experimental protocol was approved by Institute of Chinese Materia Medica, China Academy of Chinese Medical Sciences and all methods were carried out in accordance with the approved guidelines.

### Preparation of Panax ginseng and Veratrum nigrum

GS and VN were purchased from Changchun Medicinal Herbs Co. Ltd. (Jilin, China) and Anhui Tongling Medicinal Herbs Co. Ltd (Anhui, China) respectively, and identified and authenticated by Professor Liu Zhiqiang at Changchun Institute of Applied Chemistry Chinese Academy of Sciences. GS was pulverized to a fine powder and boiled twice with distilled water for 1 hour under reflux. The aqueous extracts were collected and filtered. The filtrates were then concentrated under reduced pressure at 50 °C to a concentration of 0.6 g/mL. VN was extracted by the same method and to a concentration of 0.00225 g/mL.

The samples of GS and VN for cell culture assays were prepared by extracting the powder with distilled water (three times, for 1 h each), respectively. The extracts were concentrated *in vacuo* and dissolved in DMSO (1 g/mL).

### *In vivo* studies

#### Animals and experimental design

##### Immature mice model

21-day-old female immature mice (12 ± 2 g) were purchased from the Experimental Animal Center of Academy of Military Medical Sciences (Certificate No. SCXK [Jing] 2010-0032). The mice were randomly assigned to twelve groups: control group (Con, n = 10), immature mice treated with 5 mg/kg ICI (ICI, n = 10) or 0.045 g/kg Veratrum nigrum (VN, n = 10), and immature mice treated with Panax ginseng (GS) at a daily dose of 12.0, 18.0 or 24.0 g/kg and treated with GS plus ICI or VN, (n = 10 in per group) for 7 days. According to our previous study^13^, 12 g/kg of GS is the minimum effective dose for estrogenic activity in immature mice, 0.045 g/kg VN is the clinical equivalent dosage according to “Zhong Hua Ben Cao” and is also the maximum effective dose for anti-estrogenic activity on immature mice in our repeated experiment (data is given in Fig. S1 in supplementary information). Dose calculations followed guidelines correlating dose equivalents between humans and laboratory animals on the basis of ratios of body surface area^29^. Untreated control mice received distilled water only.

##### Ovariectomized mice model

Four-week-old female KM mice (Experimental Animal Center of Academy of Military Medical Sciences, PR China, Certificate No. SCXK [Jing] 2010-0032) were maintained on normal 5-day estrous cycles as confirmed by daily vaginal epithelial cell smear testing until ovariectomy was performed. Dorsal ovariectomy was performed under general anesthesia using 30 mg/kg of pentobarbital and 500 mg/kg urethane. In sham-operated negative controls, fat near the ovary was removed. Consistent with our previous study^14^ the mice were randomly assigned to eleven groups: ovariectomy without treatment (OVX, n = 10), sham operated (sham, n = 10), ovariectomized mice treated with 0.154 mg/kg estradiol valerate (EV, n = 10) and EV plus ICI (5 mg/kg) or VN (0.045 g/kg), OVX mice were treated with GS intragastrically at a daily dose of 18.0 or 24.0 g/kg and GS plus ICI (5 mg/kg) or VN (0.045 g/kg) for 4 weeks.

All animals were maintained on a 12-h light/dark cycle under constant temperature (24 ± 2 °C) and humidity (55 ± 5%) and allowed free access to food and water. All procedures for consideration of animal welfare were reviewed and approved by the ethical committee of China Academy of Traditional Chinese Medicine (the project identification code: 20140402).

#### Analysis of tissue and serum

All Animals were sacrificed by decapitation after treatment. Blood was collected by removing the eyeball for analysis of estradiol (E_2_), follicle-stimulating hormone (FSH), and luteinizing hormone (LH) levels by enzyme-linked immunosorbent assay (ELISA) (Beijing Xinfangcheng Biotechnology, China). The uterus and adrenal gland were removed and weighed.

The left horns of the uterus and the upper portion of the vagina were stored at −80 °C for analysis by western blot or realtime PCR. The right horns of the uterus and the under portion of the vagina were fixed with 4% polyoxymethylene for 24 h. All samples were embedded in paraffin and prepared for cross sections. Sections (4-μm thick) were cut, the hydrated uterine cross sections was stained for 1–3 min in hematoxylin, washed in several changes of tap water, transferred to a saturated lithium carbonate solution, and kept for 1 min. Subsequently, they were stained in 1% Eosin Y aqueous solution, washed quickly in distilled water, and dehydrated in a graded series of ethanol and xylene. Histopathologic examination was performed under a light microscope (Leica DMI3000, Germany) and images were analyzed with Image-Pro Plus Software (Media Cybernetics Ltd., USA). The observation of uterine endometrium thickness, the number of uterine glands and vaginal epithelial layer was performed on a selected single slide in each animal.

#### Immunohistochemistry

Sections 4μm thick of uterus and vagina were used for immunohistochemical analysis. The immunohistochemistry protocol and semi-quantitative analysis were carried out as described in our previous study^7^. Rabbit anti-estrogen receptor- α polyclonal antibody (1:20, SC-542, Santa Cruz Biotechnology, Santa Cruz, CA, USA), and rabbit anti-estrogen receptor-β polyclonal antibody (1:50, ab3577, Abcam Biotechnology, Cambridge UK) were used. The Image-Pro Plus 6.0 System image analysis system was used for quantitative analysis. Five fields of view were randomly selected from each slice, and an index of positive staining was determined from the area of positive staining and the optical density. The positive index was calculated as the positive area of optical density. Immunostaining was assessed by two independent observers. First, the percentage of immunostaining was evaluated. Second, the staining intensity was recorded.

#### Western blot

ERα and ERβ in uterus and vagina were examined by western blot, the protocol and semi-quantitative analysis were carried out following our previous study^7^,^30^. The following antibodies were used: rabbit anti-estrogen receptor- α polyclonal antibody (1:200, SC-542, Santa Cruz Biotechnology), rabbit anti-estrogen receptor- β polyclonal antibody (1:1000, ab3577, Abcam Biotechnology), and rabbit anti-glyceraldehyde 3-phosphate dehydrogenase (GAPDH) monoclonal antibody (1:1000, SC-7907, Cell Signaling Technology, Danvers, MA, USA). The relative quantity of each antibody was measured by Alpha Ease FC (Fluorchem FC_2_) software. The density ratio of protein to GAPDH was calculated from the band density.

#### Real-Time Quantitative Polymerase Chain Reaction (PCR)

The RT-PCR protocol and quantitative analysis were carried out following the protocol of our previous study^14^. Gene-specific primers were used for ER α (forward: CGCCTTCTACAGGTCTAAT; reverse: GGTTCTTGTCAATGGTGC), ER β (forwar -d: CTGTGAGGTAGGAATGCGAAAC; reverse: GGTCTGGGTGATTGC GAAGA) and β-action (forward: CCTCTATGCCAACACAGTGC; reverse: CTGTAGAACGGT GTGGTCATC). The mRNA levels of ER α and ER β were normalized to the β -action mRNA level. PCR was performed as 95 °C for 10 min, followed by 40 cycles of 95 °C for 30 s and 60 °C for 1 min. The relative mRNA expression was calculated with the comparative *C t* method.

### *In vitro* studies

#### MTT assay of MCF-7, T-47D and MDA-MB-231 cell viability

The ER-positive cell line, MCF-7 and T47D cells were purchased from Cell Resource Center, IBMS, CAMS/PUMC (from the American Type Culture Collection [ATCC]) and maintained in DMEM and 10% heat-inactivated FBS (vol/vol). To minimize the effects of endogenous estrogens, cells were primed for at least 2 days in Phenol Red-free medium containing 5% charcoal-stripped FBS, and then seeded (2 × 10^3^ cells/180 μL/well) in 96-well plates. Cells were pre-incubated overnight in estrogen-depleted medium. Test samples of GS extract (20 μL at varying concentrations in DMSO), 17 β -estradiol (0.27 μg/mL), test samples with E_2_+ICI (0.27 μg/mL + 0.0061 μg/mL), GS + VN in combination, GSVN mixture at different concentrations, GS + VN mixture at varying ratios in different concentrations, and 0.1% DMSO solvent blank (the same final concentration of DMSO in test sample solutions) were added and incubated at 37 °C for 2 days. Proliferation was determined by the MTT (3-[4,5-dimethylthiazol-2-yl]-2,5-diphenyltetrazolium) bromide assay at 490 nm. Percent growth induction was calculated as a percentage of the average response of the DMSO control samples. Results reported are the mean ± standard deviation of four replicate determinations from a representative assay^31^,^32^.

The ER-negative cell line, MDA-MB-231 cell was purchased from ATCC, maintained in DMEM and 10% heat-inactivated FBS (vol/vol) and seeded (6 × 10^3^ cells/180 L/well) in 96-well plates. Test samples and method as described above.

#### Measurement of ERα and ER β expressions

MCF-7 cells were depleted of estrogen, as described above, pre-incubated overnight in estrogen-depleted medium at a density of 1 × 10^6^ cells per dish, and then treated with GS (100~1 μg/mL)/17β-estradiol (0.01 μM) or in combination with ICI (0.01 μM)/ VN (10~0.1 μg/mL), and 0.1% DMSO treatment as negative control. All cells were incubated for 48 h and were harvested protein. The Western blot protocol and semi-quantitative analysis were carried out as follows: ER α antibody (dilution 1/200, SC-542 Santa Cruz Biotechnology, CA) and ER β antibody (dilution 1/1000, ab3577, Abcam Biotechnology) were used and GAPDH antibody (dilution 1:1000, SC-7907, Cell Signaling Technology, Boston, Massachusetts, USA) was used as internal control. All experiments were done in triplicate. Mean normalized gene expression ± standard deviation was calculated from independent experiments.

#### Measurement of ER α and ERβ mRNA expressions

MCF-7 cells were treated as described above. The RT-PCR protocol and quantitative analysis were carried out following the protocol of our previous study^14^. Gene-specific primers were used for ERα (forward: 5′–CCT CCC TGA ACT TGC AGT AA–3′; reverse: 5′–CCT GCT CCT TTC AAC TAC CA–3′), ERβ (forward: 5′ – CGA CAA GGA GTT GGT ACA CAT GA–3′; reverse: 5′–CCA AGA GCC GCA CTT GGT–3′) and GAPDH (forward: 5′–GTC AGT GGT GGA CCT GAC CT–3′; reverse: 5′–AAA GGT GGA GGA GTG GGT GT–3′). The messenger (m) RNA levels of ERα and ERβ were normalized to the GAPDH mRNA levels. PCR was performed at 95 °C for 10 min, followed by 40 cycles of 95 °C for 30 sec, and 64 °C for 1 min. The relative mRNA expression was calculated by the comparative threshold cycle (*Ct*) method.

#### Statistical analysis

The SPSS software version 11.0 for Windows (SPSS Inc, Chicago, IL, USA) was used for statistical analysis. All data were expressed as mean ± standard deviation and were analyzed by one-way analysis of variance (ANOVA) followed by least significant difference (LSD) or Dunnett’s T3 test. Differences were considered statistically significant when p was less than 0.05.

## Additional Information

**How to cite this article**: Xu, Y. *et al*. Effect of the Interaction of Veratrum Nigrum with Panax Ginseng on Estrogenic Activity *In Vivo* and *In Vitro. Sci. Rep.* **6**, 26924; doi: 10.1038/srep26924 (2016).

## Supplementary Material

Supplementary Information

## Figures and Tables

**Table 1:** Quantitative date of histological feature in uterus and vagina of immature mice.

**Group**	**Uterus endometrial thickness(μM)**	**Uterus endometrial glands numbers**	**Vagina epithelium cell layers**	**Vaginal thelium thickness(μM)**
Con	65.95 ± 19.29	8.50 ± 2.50	7.60 ± 2.24	10.94 ± 3.63
ICI	35.22 ± 9.54^***^	5.30 ± 2.76^*^	3.90 ± 2.17^**^	5.47 ± 2.12^**^
VN	42.78 ± 17.07^*^	8.00 ± 3.00	5.20 ± 1.89*	7.81 ± 1.47*
GS12	83.47 ± 21.37	11.30 ± 3.72	7.80 ± 2.96*	17.07 ± 5.92^*^
GS12+ICI	33.54 ± 8.80^###^	5.70 ± 2.97^##^	3.80 ± 2.27^##^	7.27 ± 2.09^###^
GS12+VN	43.30 ± 21.35^△△^	7.10 ± 3.48^△^	4.80 ± 2.86^△^	9.97 ± 2.53^△△^
GS18	111.88 ± 22.84^***^	15.10 ± 4.16^***^	11.40 ± 3.07^**^	22.87 ± 6.19^***^
GS18+ICI	43.62 ± 26.97^###^	8.80 ± 5.58^#^	6.20 ± 2.18^###^	9.60 ± 3.14^###^
GS18+VN	49.15 ± 25.67^△△△^	8.20 ± 3.79^△△^	7.30 ± 2.87^△△^	11.07 ± 3.11^△△△^
GS24	101.11 ± 22.61^**^	14.10 ± 4.21^**^	10.10 ± 3.91^*^	20.01 ± 6.95^**^
GS24+ICI	45.63 ± 16.48^###^	7.70 ± 4.65^##^	5.90 ± 3.81^###^	10.15 ± 2.20^###^
GS24+VN	64.16 ± 24.25^△△^	8.50 ± 4.48^△^	8.10 ± 2.21^△^	12.90 ± 2.61^△^

***P < 0.001, **P < 0.01 and *P < 0.05, compared with the Con or OVX group; ^###^*P* < 0.001 ^##^*P* < 0.01 and ^#^*P* < 0.05, compared with the GS group or EV group; ^∆∆∆^*P* < 0.001, ^∆∆^*P* < 0.01 and ^∆^*P* < 0.05, compared with the GS group or EV group.

**Table 2:** Quantitative date of histological feature in uterus and vagina of OVX mice.

**Group**	**Uterus endometrial thickness(μM)**	**Uterus endometrial glands numbers**	**Vagina epithelium cell layers**	**Vaginal epithelium thickness(μM)**
Sham	243.10 ± 34.26	27.90 ± 3.42	8.40 ± 2.29	25.67 ± 5.36
OVX	106.41 ± 26.78^▲▲▲^	9.40 ± 2.91^▲▲▲^	2.70 ± 1.55^▲▲▲^	4.98 ± 3.90^▲▲▲^
EV	228.25 ± 47.93^***^	25.10 ± 2.59^***^	8.60 ± 2.42^***^	25.30 ± 4.10^***^
EV+ICI	166.63 ± 36.28^**#**^	19.20 ± 3.34^**###**^	5.50 ± 1.68^##^	18.45 ± 4.13^##^
EV+VN	179.68 ± 34.48^**△**^	22.20 ± 2.89^**△**^	5.40 ± 1.74^△△^	20.05 ± 4.46^△^
GS18	184.27 ± 76.29^**^	17.20 ± 5.83^**^	6.60 ± 2.25^***^	18.62 ± 7.86^***^
GS18+ICI	118.28 ± 18.18^**#**^	13.80 ± 3.76	3.80 ± 1.25^##^	10.08 ± 3.67^##^
GS18+VN	158.35 ± 29.24	12.30 ± 3.10^△^	4.70 ± 1.42^△^	10.63 ± 2.65^△△^
GS24	206.62 ± 72.84^**^	22.20 ± 8.65^***^	7.80 ± 2.71^***^	20.89 ± 10.34^***^
GS24+ICI	133.26 ± 16.14^**#**^	15.30 ± 3.69^**#**^	3.90 ± 2.17^##^	11.79 ± 4.14^#^
GS24+VN	169.16 ± 35.16^**△**^	15.20 ± 4.64^**△**^	4.50 ± 2.16^△^	13.81 ± 3.69

***P < 0.001, **P < 0.01 and *P < 0.05, compared with the OVX group; ^###^*P* < 0.001 ^##^*P* < 0.01 and ^#^*P* < 0.05, compared with the GS group or EV group; ^∆∆∆^*P* < 0.001, ^∆∆^*P* < 0.01 and ^∆^*P* < 0.05, compared with the GS group or EV group; ^▴▴▴^*P* < 0.001, compared with the Sham group.
