# Corrigendum: Effect of the Interaction of Veratrum Nigrum with Panax Ginseng on Estrogenic Activity *In Vivo* and *In Vitro*

**DOI:** 10.1038/srep45946

**Published:** 2017-04-05

**Authors:** Ying Xu, Jie Ding, Jin-na An, Ya-kun Qu, Xin Li, Xiao-ping Ma, Yi-min Zhang, Guo-jing Dai, Na Lin

Scientific Reports 6: Article number: 2692410.1038/srep26924; published online: 05 27 2016; updated: 04 05 2017

The original version of this Article contained an error in the reference list, where reference 5 was incorrectly listed as “O’Hara, M., Kiefer, D., Farrell, K. & Kemper, K. *A review of 12 commonly used medicinal herbs. Arch Fam Med*. **7**, 523–536 (1998)”. The correct reference 5 appears below.

This has now been corrected in the HTML and PDF versions of this Article.

**Reference**

Park, J. *et al*. Interaction of Veratrum nigrum with Panax ginseng against obesity: A Sang-ban Relationship. *Evid-based Compl Alt*. **10**, 732126 (2013).

