# Supplementary material for: Effect of the Interaction of Veratrum Nigrum with Panax Ginseng on Estrogenic Activity In Vivo and In Vitro
Source: Sci Rep. 2016 May 27;6:26924. doi: 10.1038/srep26924 (PMC4882595; doi:10.1038/srep26924)
Supplement: Supplementary Information [file srep26924-s1.pdf]

Effect of the Interaction of Veratrum Nigrum with Panax Ginseng on Estrogenic Activity *In Vivo* and *In Vitro*

---Treatment with Veratrum Nigrum Exerts Anti-estrogenic Activity *In Vivo* and *In Vitro*

Ying Xu[#*]*, Jie Ding*[#]*, Jin-na An*[#]*, Ya-kun Qu, Xin Li, Xiao-ping Ma, Yi-Min Zhang, Guo-jing Dai, Na Lin*[*]

#These authors contribute equally to this study.

**Corresponding author: Prof. Na LIN & Ying Xu*

Abstract

To characterize anti-estrogenic effects of Veratrum nigrum (VN) using immature mice together with *in vitro* studies for further molecular characterization. Immature mice were treated with VN at doses of 0.03, 0.045, 0.06 and 0.09 g/kg for 7 days. VN at 0.045 g/kg significantly decreased serum estradiol level, inhibited the growth and development of uterus and vagina, and down-regulated ERα and ERβ expression in reproductive tissues. VN had an inhibited effect on MCF-7 cell viability. VN significantly inhibited luciferase expression from the ERα/β-estrogen response element (ERE) luciferase reporter. This study demonstrated VN exerts anti-estrogenic effects on reproductive tissues in immature mice by inhibited biosynthesis of estrogen in circulation, down-regulating ERs and maybe through ER-ERE-dependent pathway.

Results and Discussion

Effect of Veratrum nigrum (VN) on uterine weight and serum E_2

Uterine weight was significantly decreased in immature mice treated with ICI, 182780 (P < 0.001). Treatment of immature mice with VN had modest inhibited effects on uterine weight, the medium dose 0.045 g/kg treatment resulted significant differences compared with untreated controls (P <0.05). Additional, treatment of immature mice with VN at 0.045 g/kg or with ICI, significantly decreased circulating E_2 level compared to those of untreated immature mice (P < 0.010 or 0.001) (Fig. S1), suggesting that VN at 0.045 g/kg has significant anti-estrogenic activity. These data prompted further studies to elucidate the activity and mechanism of VN at tat 0.045 g/kg

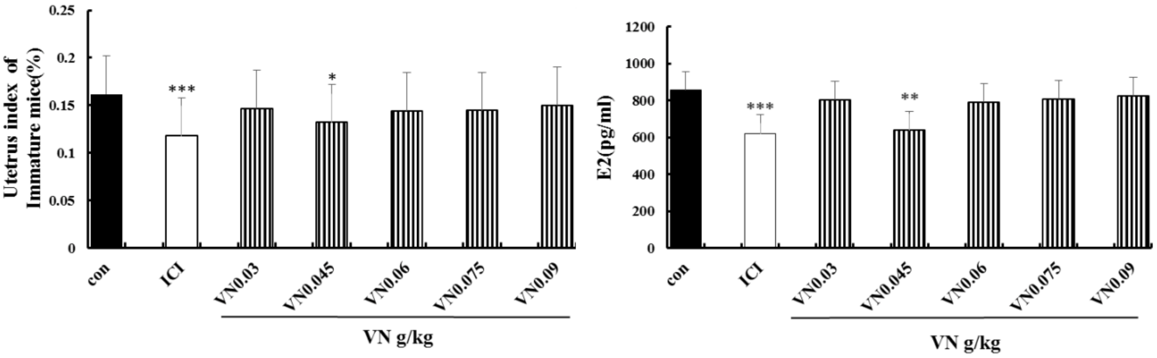

Fig.S1 The effects of Veratrum nigrum (VN) on uterine weight and serum $E_{2.}$ The uterine weights and serum E_2 of immature mice were measured at the end of the 7-day treatment period.. Data are the mean and standard deviation (SD) of samples from 10 mice. P values are for the one-way analysis of variance (ANOVA) comparing the treatment group with untreated mice. (***) P <0.001, (**) P < 0.01, and (*) P < 0.05 compared with the Con.

Effect of VN on histopathology of uterus and vagina

Histological analysis of uterine sections revealed treatment of immature mice with ICI or VN 0.045 g/kg substantially changed uterine morphology (Fig.S2), as indicated by thin of the uterine endometrium, decreased number of glands, and narrowed glandular cavities compared with untreated controls. Fig.S2 shows microscopic preparations of representative vagina from one animal per treatment

group, compared with untreated immature mice, treatment with ICI or VN 0.045 g/kg decreased epithelial thickness and also the number of cell layers. Taken together, these studies provide evidences that VN has anti-estrogenic activity, comparable to that of classical estrogen receptor antagonist ICI, 182780.

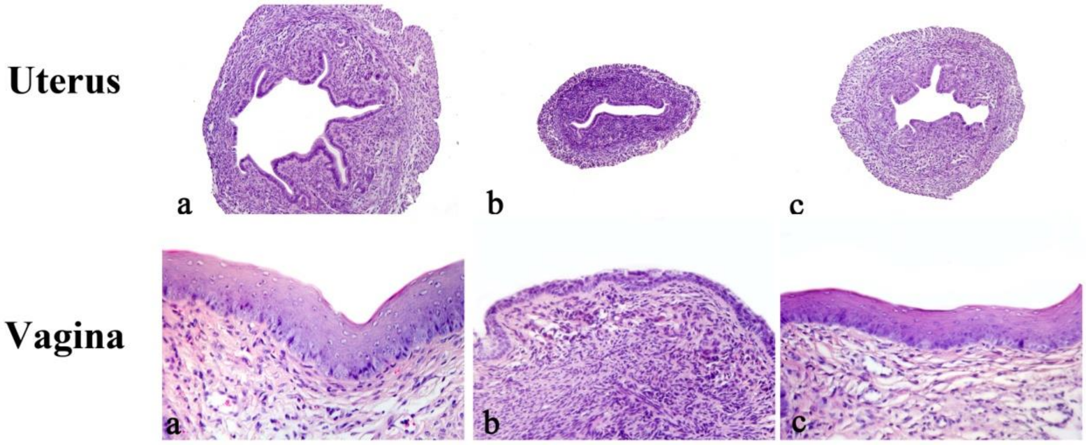

Fig.S2 The effects of VN on histopathology of uterus and vagina. Representative photomicrographs taken at 200-X magnification of uterine and 400-X magnification of vaginal sections. The treatment groups are shown: (a) control group; (b) treated with ICI; (c) treated with VN at 0.045 g/kg.

Effect of VN on ERs expression by immunohistochemistry in uterus and vagina

The expressions of ERα and ERβ in the uterus and vagina from each group and quantitative analysis are shown in Fig. S3. Treatment with either ICI or VN induced clear and comparable down-regulation of ERα and ERβ in the uterus and vagina compared with Con group. ERs in uterus were expressed in similar cell types in the ICI treated or VN treated groups, namely in the epithelial cells of the endometrium, interstitial cells and smooth muscle cells. ERs in vagina were expressed in vaginal epithelium cells of squamous cell and smooth muscle cells.

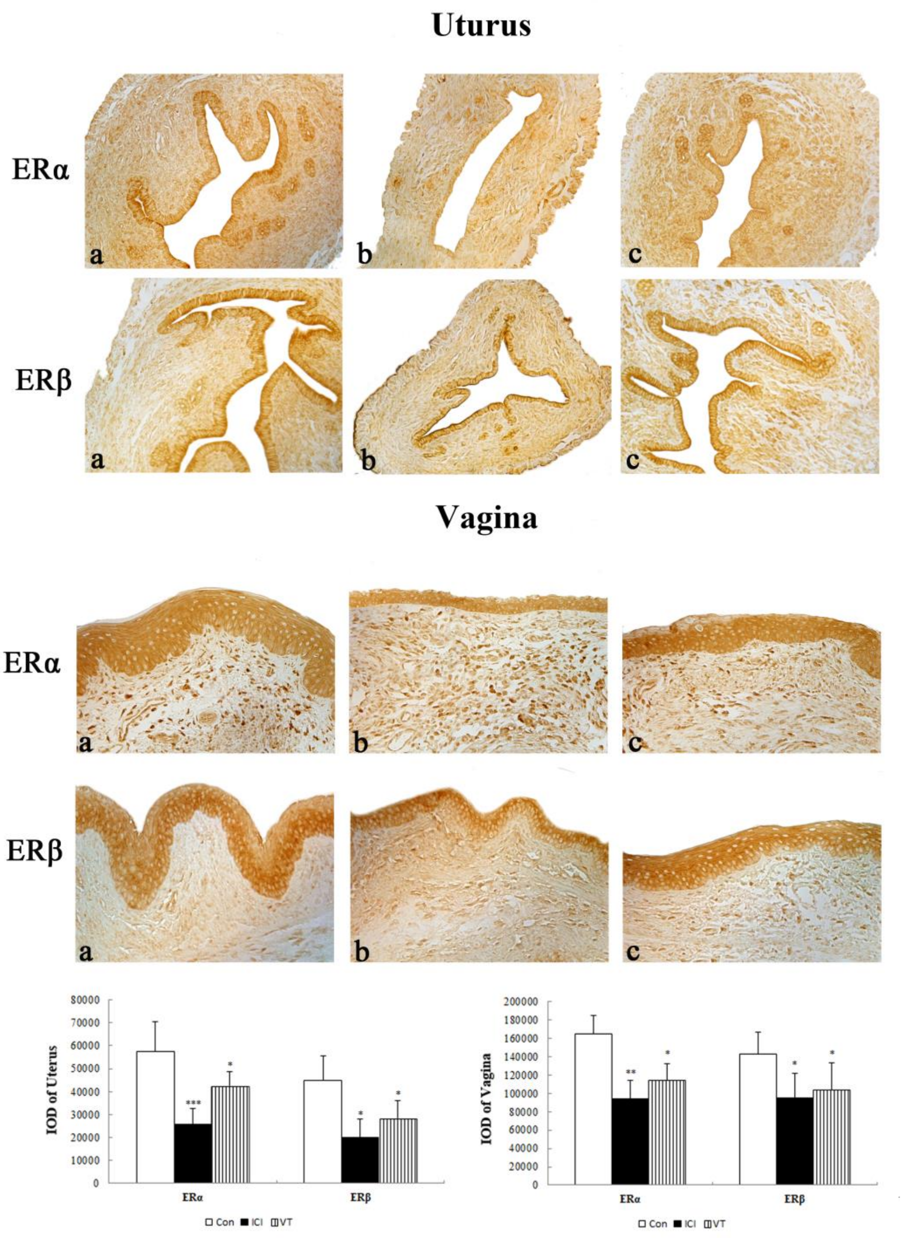

Fig.S3 The effects of VN on the expressions of estrogen receptor (ER) α and ER β in the uterus and vagina. histopathology of uterus and vagina. Representative photomicrographs taken at 200-X magnification of uterine and 400-X magnification of vaginal sections. The treatment groups are shown: (a) control group; (b) treated with ICI; (c) treated with VN at 0.045 g/kg.

VN inhibited MCF-7 cell viability

To investigate the molecular basis of VN activity in more detail, we used MCF-7 human breast cancer cells as a model because it is estrogen receptor positive cell. 0.01~100 μg/mL VN and 0.0061 μg/mL both inhibited MCF cell viability compared

with DMSO control ($P < 0.05$ or 0.01). (Fig.S4), demonstrating the anti-estrogenic activity of VN.

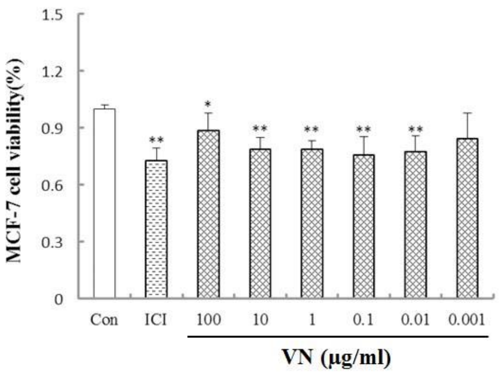

Fig.S4 The effect of VN on the viability of MCF-7 cell. Results are expressed relative to growth of cells treated with 0.1% DMSO. Data are the mean and standard deviation of quadruplicate analyses, expressed relative to that of treatment with 0.1% DMSO. (*) $P <0.05$ and (**) $P < 0.01$ compared with DMSO.

VN inhibited both ERα and ERβtranscriptional activity.

HEK 293 cells that had been stably transfected with the hERα/β -ERE-luciferase plasmid were used to measure the formation of functional hERα/β -ERE complexes in response to treatment with the VN extracts. Results are expressed relative to expression in DMSO-treated cells. ICI or VN significantly decreased both ERα and ERβ -ERE luciferase activity ($P < 0.05$, 0.01 or 0.001). Moreover, Fig.S5 showed that ICI or VN at any doses significantly decreased ERα/β -ERE-luciferase activity induced by 17 β-estradiol. These results shown VN had a dual action on ERα and ERβ.

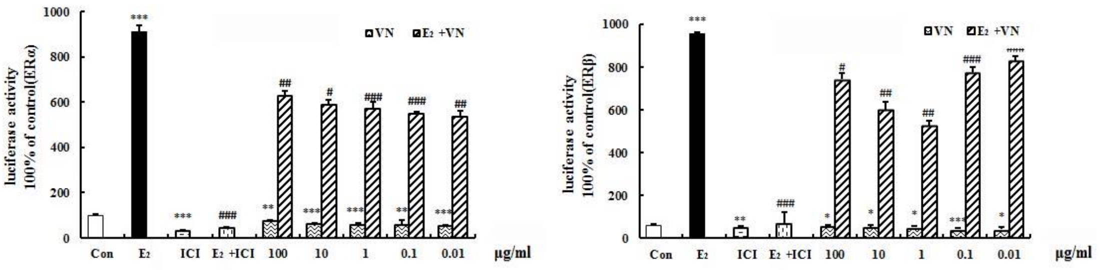

Fig.S5 Activity of VN on estrogen receptor (ER) α/β –estrogen response element (ERE) luciferases reporter gene expression. Data are the mean ± standard deviation of quadruplicate analyses, expressed relative to that of treatment with 0.1% DMSO. *p < 0.05, **p < 0.01 compared to DMSO; # p < 0.05, ### p < 0.001, compared to 0.27μg/mL E_2 .

Conclusion:

Our findings suggest that VN had anti-estrogenic effect on reproductive tissues in immature mice by inhibited biosynthesis of estrogen in circulation, down-regulating ERs and maybe through ER-ERE-dependent pathway.

Methods

In vivo studies

Immature mice model: 21-day-old female immature mice (12 ± 2 g) were purchased from the Experimental Animal Center of Academy of Military Medical Sciences (Certificate No. SCXK [Jing] 2010-0032). The mice were randomly assigned to seven groups: control group (Con, n=10), immature mice treated with 5 mg/kg ICI (ICI, n=10), and immature mice treated with Panax ginseng (GS) at a daily dose of 0.03, 0.045, 0.06 and 0.09 g/kg for 7 days. 0.045 g/kg VN is the clinical equivalent dosage according to “Zhong Hua Ben Cao” .Untreated control mice received distilled water only.

All Animals were sacrificed by decapitation after treatment. Blood was collected by removing the eyeball for analysis of estradiol (E_2). Uterus were weighted, the left horns of the uterus and the upper portion of the vagina were fixed with 4% polyoxymethylene for histopathology by HE and ERs expression by immunohistochemistry. The methods as described in main manuscript.

***In vitro* studies**

MTT assay of MCF-7 cell viability

The ER-positive cell line, MCF-7 cell line was from ATCC and maintained in DMEM and 10% heat-inactivated FBS (vol/vol). To minimize the effects of endogenous estrogens, cells were primed for at least 2 days in Phenol Red-free medium containing 5% charcoal-stripped FBS, and then seeded (8×10^3 cells/180 μL/well) in 96-well plates. Cells were pre-incubated overnight in estrogen-depleted medium. Test samples of VN extract (20 μL at varying concentrations in DMSO), ICI（0.0061 μg/mL）, and 0.1% DMSO solvent blank (the same final concentration of DMSO in test sample solutions) were added and incubated at 37°C for 2 days. The detecting and analysis method as described in main manuscript.

Transfection and reporter assay of estrogen receptor-subtype selectivity

HEK 293 cells were stably transfected with human estrogen receptor α /β (hER α /β) and the estrogen response element (ERE) plasmid (kindly provided by Professor Yung-Chi Cheng, Yale University), and the luciferase reporter assay system from Promega (WI, USA) was used to evaluate the formation of functional ER α /β -ERE

complexes. The cells were maintained and primed to minimize the effects of endogenous estrogens as described above and then seeded (1×10^5 cells/100 μL/well) in 96-well plates. The test samples with or without 17β-estradiol were added to three replicate wells, as described for the MTT assay of MCF-7 cell proliferation, and was incubated for 24 h. Finally, the growth medium was carefully removed and 50 μL of lysis buffer per well was added, and the plate was rocked for 15 min. Twenty microliters of the detached cell solution was then transferred to a white micro well plate. Luciferase assay reagent (50 μL) was added to each well, and luciferase activity was measured immediately. Activity of the luciferase reporter gene was expressed relative to the DMSO control. Results reported are the mean ± standard deviation of three replicate determinations from a representative assay.
